# Intracorneal Rings (INTACS SK) Might be Beneficial in Keratoconus; A Prospective Nonrandomized Study

**Published:** 2013

**Authors:** Tarek A. Ibrahim, Osama Elmor

**Affiliations:** Eye Consultants, Dubai Healthcare City, Dubai, UAE

**Keywords:** Intracorneal Ring, Keratoconus, Pachymetry, Uncorrected Visual Acuity (UCVA), Best Corrected Visual Acuity (BCVA)

## Abstract

In order to determine the effect of intracorneal rings (Intacs SK), when implanted in keratoconic patients, on corneal curvature, Uncorrected Visual Acuity (UCVA), Best Corrected Visual Acuity (BCVA) and on the progression of the cone through three years follow-up period. In this prospective nonrandomized study 114 eyes of 71 keratoconic patients (38 females and 33 males) were implanted with Intacs SK. Incisions were always made in the steep meridian. UCVA, BCVA, Corneal Topography (TMS) were measured pre and postoperatively and at intervals of 1, 3, 6 & 12 months then yearly for 3 consecutive years.

Preoperative mean k-reading was 52.53 and 48.18, 49.56, 49.17, 48.51, 48.15 & 48.01 at 1, 3, 6, 12, 24 & 36 months postoperatively (P‹0.01). In terms of UCVA, 15.64% of patients gained more than 3 lines and 69.73% gained 1-3 lines with a total of 85.37% of patients gaining lines compared to their preoperative UCVA (P‹0.01) while 14.63% of cases did not gain any lines at 1 month postoperative. At three months postoperatively, 12.64% gained more than 3 lines, 71.15% gained 1-3 lines with a total of 83.79% while 16.21% did not gain any lines. Three years postoperative 11.82% of cases gained more than 3 lines, 73.23% gained 1-3 lines with a total of 85.05% while 14.95% did not gain any lines (P‹0.01). With regard to BCVA, 19.73% gained more than 3 lines, 68.26% gained 1-3 lines with a total of 87.99% of cases gaining lines compared to their preoperative BCVA (P‹0.01) while 12.01% did not gained any lines at 1 month postoperative. At three months postoperatively, 14.96% gained more than 3 lines, 70.19% gained 1-3 lines with a total of 85.15% while 14.85% did not gain any lines. Three years postoperative, 12.17% gained more than 3 lines, 71.78% gained 1-3 lines with a total of 83.95% (P‹0.01) while 16.05% did not gain any lines. No eyes lost any lines as it pertained to UCVA & BCVA. Despite the fluctuation of k-readings, UCVA and BCVA in the first 3 months, which may represent the time needed to stabilize the cone, UCVA and BCVA were improved and maintained throughout the study. Patient selection remains the key point for the success of intacs in keratoconic patients.

## INTRODUCTION

In the past few years, computer technology and biotechnology have had a major impact on improving our understanding of keratoconus. Keratoconus, a Greek derivate, means conical cornea and may be defined as a progressive non-inflammatory ectatic corneal disorders [1-3]. The treatment is mainly focused on optical reason in early and moderate cases; however, surgery is required in advanced cones. Spectacles insufficiently compensate for the optical effects produced by the irregular astigmatism. Rigid gas permeable contact lenses give satisfactory visual results in most cases, however, there are obvious practical and clinical concerns (i.e., contact lens intolerance and contact lens related problems) remain. Adequate tear exchanges, optical clearance and gas permeability of the system are essential to provide enough oxygen and to avoid hypoxic damage and scarring of the apex of the diseased cornea [4-6]. 

Lamellar or penetrating keratoplasties are, from the surgeon’s point of view, a radical solution, because functional recovery following transplantation is usually long, in some cases more than one year. Furthermore, there may be troublesome postoperative ametropia. The need for additional correction often arises (eg. contact lenses, LASIK, PRK, relaxing or wedge incisions or toric phakic IOLs) to correct post transplantation refractive errors. Moreover, some sight-threatening complications must be considered which may be related to graft survival, which, although varies between patients but decreases with time. Graft rejection and endothelial cell failure may also occur. Recurrences remain a main threat, with the peripheral ring of the recipient cornea being the potential source of further problems. About 56% of keratoconic eyes successfully treated with penetrating keratoplasty show progressive development of astigmatism 10 to15 years after surgery. Lastly, one big problem most surgeons face is the poor availability of optimum quality donor tissue especially in countries where the culture of donation is low [1,7-8].

The majority of keratoconic patients are young and in the prime of their lives. They seek a minimal risk solution that gives high quality of vision, rapid rehabilitation and minimal discomfort and pain [9-11]. Generally, well-informed patients are reluctant to undergo corneal transplantation, while surgeons should consider it as the last resort. In fact, patients and surgeons should be interested in a more conservative alternative, thereby delaying the need for a cornea graft [7-10].

Intacs inserts are currently approved by the U.S. Food and Drug Administration (FDA) and by the Communauté Européene for use in the reduction or elimination of myopia and astigmatism. They are reported to be an effective modality for the treatment of keratoconus and to stabilize ectasia resulting from keratorefractive surgery or other causes [11-14]. 

The purpose of our study was to evaluate the effect of Intacs intrastromal rings when implanted in keratonic patients on corneal curvature, uncorrected visual acuity (UCVA), best corrected visual acuity (BCVA), lower and higher order aberrations, and on the progression of the cone.

## METHODS

We implanted 114 eyes of 71 keratokonic patients with Intacs SK (Addition Technology, Des Plaines, III). Thirty eight (38) were females and 33 were males with a follow-up period of 3 years. Preoperative and postoperative assessment included: slit-lamp examination, BCAV and UCVA (decimal chart), corneal topography (Topographic Modeling System “TMS-2”; Tomey, New York, NY), corneal thickness using ultrasonic pachymetry (DGH-1000, DGH Technology, Inc. Exton, Pa) and Optical Path Difference (OPD) Scan (Nidek, Tokyo, Japan) where Zernike graph with the total and differential ocular aberrations were compared.

Inclusion criteria involved: keratoconus stage I-II based on Amsler-Krumeich classification, with clear central cornea, best corrected visual acuity more than 0.2, central pachymetry more than 400µm, and intolerance to contact lens wear. 

**Figure 1:**
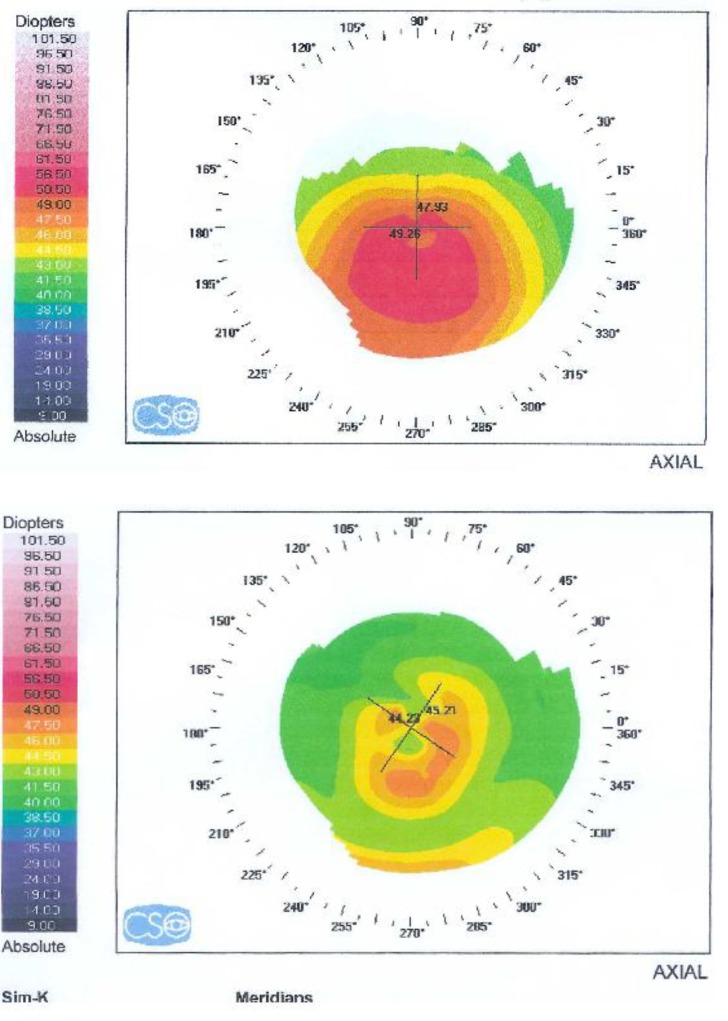
Shows parameter in preoperative cornea of the left eye (top) and three Months after Implantation in the same patient (below).

Surgical procedure was performed under topical anesthesia (0.4 Benoxinate) by the same surgeon with the Intralase Femtosecond laser using a modified technique of the described by Addition Technology in all cases. The only modification was that the tunnel size was adjusted to (5.9-7.1 mm) instead of (6-7 mm) as advised by the company. The incision was always made in the steep meridian. Centration was made on the geometric center of the cornea. Twenty minutes postoperatively, slit-lamp examination was done for stromal depth estimation and recording of each implant and then patient was discharged home. Patients were examined 1st day, 7th day mainly for postoperative infection and patient compliance, then at 1st, 3rd, 6th, 12th months, 2 and 3 years postoperatively. Sutures were removed on a case by case basis but not less than 8 weeks postoperatively (Figure 1). 

## RESULTS

**Figure 2:**
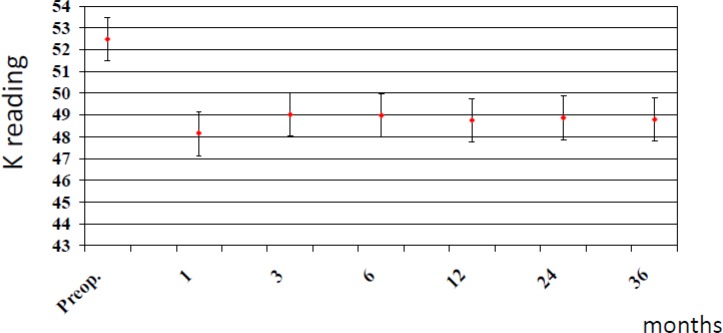
Post-operative mean k reading

The average age of patients was 28.9 ± 6.2 years. Preoperative mean keratometric reading was 52.53 (range, 47.00 D to 55.60 D) and 48.18, 49.56, 49.17, 48.51, 48.15, 48.01, 48.08 and 48.05 at 1, 3, 6, 12 months and 2 and 3 years postoperatively, respectively (P<0.01). Minimum and maximum keratometry were reduced at all postoperative time points after placement of rings (Figure 2). 

In terms of UCVA, 15.64% of patients gained more than 3 lines, 69.73% gained 1-3 lines with a total of 85.37% of patients gaining lines compared with their preoperative UCVA while 14.63% of cases did not gain any lines at 1 month postoperative. Three months postoperative, 12.64% gained more than 3 lines, 71.15% gained 1-3 lines with a total of 84.79% while 16.21% did not gain any lines. Six month postoperative, 12.3% gained more than 3 lines, 72.1% gained 1-3 lines with a total of 84.4% while 15.6% did not gain any lines. One year postoperative, 12.19% gained more than 3 lines, 72.90% gained 1-3 lines with a total of 85.11% while 14.90% did not gain any lines. Two years post-operative, 11.8% gained more than 3 lines, 73% gained 1-3 lines with a total of 84.4% while 15.2% did not gain any line. Three years postoperative 11.82% gained more than 3 lines, 73.23% gained 1-3 lines with a total of 85.05% while 14.95% did not gain any lines (P<0.01). 

In terms of BCVA, 19.73% of patients gained more than 3 lines, 68.26% of patients gained 1-3 lines with a total of 87.99% of cases gaining lines compared with their preoperative BCVA while 12.01% did not gain any lines at 1 month postoperative. At three months postoperative, 14.96% gained more than 3 lines, 70.19% gained 1-3 lines with a total of 85.15% while 14.85% did not gain any lines. Six months postoperative, 14.10% gained more than 3 lines, 71.50% gained 1-3 lines with a total of 85.60% while 14.40% did not gain any lines. One year postoperative 13.8% gained more than 3 lines, 71.3% gained 1-3 lines with a total of 85.1% while 14.9% did not gain any lines. Two years post-operative, 12.8% gained more than 3 lines, 73.7% gained 1-3 lines with a total of 86.5% while 13.5% did not gain any line. Three years postoperative, 12.17% gained more than 3 lines, 71.78% gained 1-3 lines with a total of 83.95% while 16.05% did not gain any lines. 

Corneal topography illustrated the anterior corneal surface changes induced by Intacs inserts. Topographic surface quality indices suggested that surface regularity improved and surface asymmetry was reduced with treatment (Figure 1, below). Extent of corneal ectasia and height of the cone were improved in all cases. Postoperative minimum simulated keratometric readings were approximately 4 diopters less than the baseline. 

**Figure 3:**
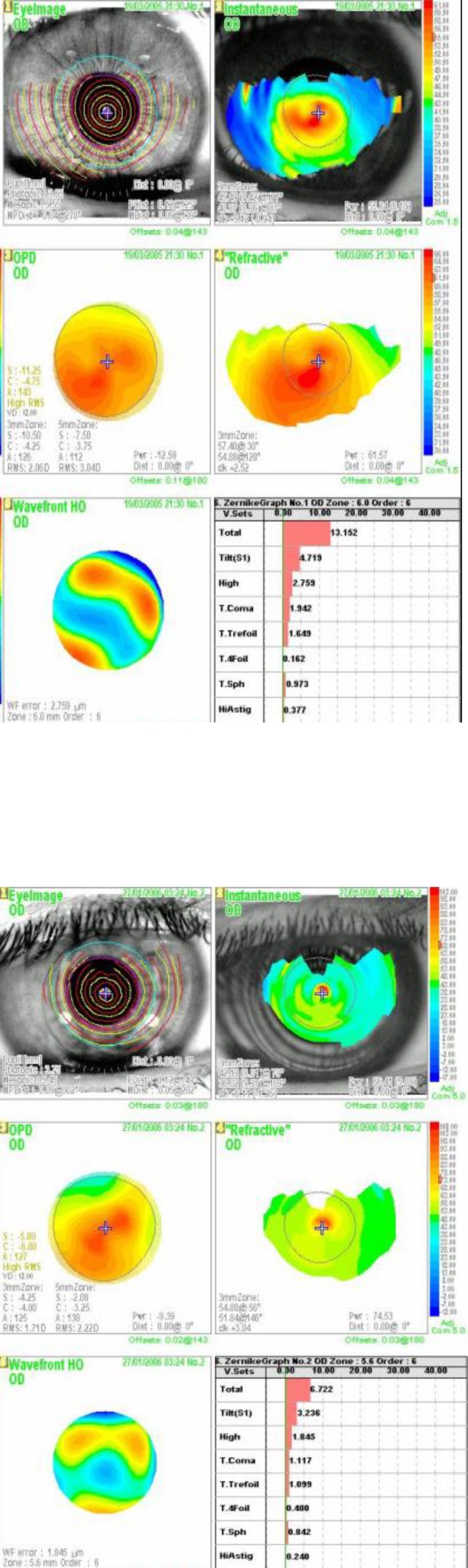
Wave-front technology, as measured by the OPD Scan. Pre Intacs (top figure) versus post intacs view (below figure).

Wave-front technology (Figure 3), as measured by Optical Path Difference (OPD) scan, revealed a decrease in both lower and higher order aberrations. It is worth mentioning, here, that some patients who did not have quantitative vision improvement post-surgery reported improvement in night vision in the form of decrease in glare and subsequently improvement in vision quality. This may be attributed to the decrease in their high order aberration after surgery; however those findings were only subjective qualitative observation. 

There were no intraoperative complications, except for one case where suction was impossible due to subconjunctival hemorrhage produced by the fixation forceps during incision making. This case was postponed and redone after the subconjunctival hemorrhage subsided and sustained no complication. Mild corneal deposits occurred mainly at the superior edge of the segments in some cases but they disappeared by the third month and necessitated no special treatment.

**Figure 4:**
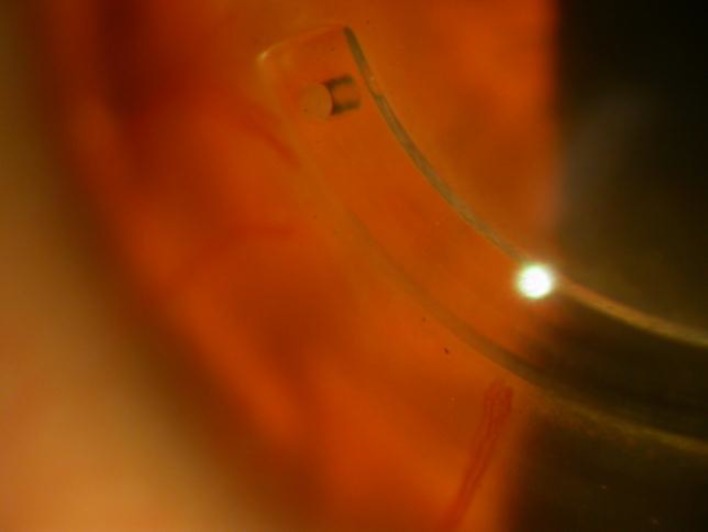
Corneal neovascularization appeared in a 18 months month post-Intacs in a patient who used soft contact lens

One case of corneal neovascularization appeared in 18 month post-Intac patient who used soft contact lens. Patient stopped contact lens wear and was treated with a steroid. Fortunately, the neovessels did not progress and the patient is being followed up every 3 months for any progression (Figure 4). 

Two rings were explanted, one due to direct ocular trauma 6 weeks post-surgery where the patient presented with the tube half extruded and the other due to continued progression of the cone one year post surgery. Both Intacs were easily removed under topical anesthesia through the original incision. Both patients underwent a successful deep anterior lamellar keratoplasty 4 weeks post-Intacs removal. No eyes lost any lines with regard to their preoperative UCVA & BCVA.

## DISCUSSION

It is imperative to set clear goals before using intracorneal ring segments for the management of keratoconus. Since Intacs are not used to eliminate the disease but to decrease corneal abnormality, our primary goals were to convert contact lens intolerant patients to contact lens tolerant ones, and delay or stop the progression of the disease and thus obviate the need for corneal transplant. A secondary goal was to decrease corneal surface irregularity thus allowing for the transition from rigid to soft contact lenses [14-16].

In our study, corneal topographic maps qualitatively demonstrated reduction of corneal ectasia and improved cone height in all cases. The flattening effect, together with decreased corneal surface irregularities, enabled soft contact lens use in most of our patients. We noticed fluctuations in K-readings during the first three postoperative months. This may represent the time needed for the tubes to stabilize the cone after which the curve becomes steady and stable.

Intracorneal ring segment implantation decreased the incidence of lower and higher order aberrations, as measured by the OPD scan. Some patients who did not have post-surgical quantitative vision improvement reported decreased glare and improved vision quality especially by night, which may be attributable to decreased higher order aberration [17-20]. 

Several studies have shown that the use of Intacs for early or moderate keratoconus achieves reshaping the abnormal without removing corneal tissue or touching the central cornea. The central cornea in these patients remains clear despite borderline corneal thickness [8,10,16,21]. 

Stability is a critical issue for any surgical intervention, and postoperative results demonstrated that spherical and astigmatic errors together with uncorrected, best corrected visual acuity and keratometric readings improved in about 85% of eyes over preoperative baseline measures and remained stable over the 5-year follow up period [9]. In fact, time is in favor of Intacs as one can see the cone becoming smaller with time. 

Intacs inserts seemed to be a minimally invasive technique for effectively reducing the corneal steepening and corneal surface irregularities associated with keratoconus and thus improving visual quality [12]. Some authors extended the use of Intacs to smooth the corneal surface post complicated refractive surgery and to support the cornea in pellucid marginal degeneration [22-30].

We adopted traditional mechanical technique for fashioning the channels, which carries the possible hazards of epithelial defects at the keratotomy site; anterior and posterior perforations during channel creation; extension of the incision toward the central visual axis or toward the limbus; shallow placement and/or uneven placement of Intacs segments; infectious keratitis, introduction of the epithelial cells into the channel during channel dissection; asymmetric placement, persisting incisional gap, and corneal stromal edema around the incision and channel from surgical manipulation [31-35]. Fortunately, we met none of these complications; although we did have difficulty in placing the suction ring in one case.

Some authors are not convinced that Intacs prevent progression of the cone or ultimately eliminate the need for keratoplasty. In our study, cone progression occurred in only one eye (0.53%) and Intacs were removed. This uncertainty of stopping cone progression in the other studies may be attributed to patient selection criteria. Most of these series were done on advanced cones in patients referred for penetrating keratoplasty. In these cases, the process of corneal decompensation had already started and could not be stopped with Intacs so the cone will definitely continue to progress [10, 16, 36].

Should keratoplasty become necessary, we advise removing Intacs prior to performing keratoplasty as a separate first step. Conducting keratoplasty simultaneous to Intacs removal may induce undesirable postoperative astigmatism [8,11,36]. 

Neovascularization may occur in long-term contact lens wearers and/or limbal neovessels. We had one case of corneal neovascularization (0.53%) (Figure 4), which responded to discontinuance of contact lens wear and topical prednisolone acetate. When the incision is performed on the temporal meridian, vessels are very uncommon. Our incision was made in the steep meridian, so we advise considering corneal dimensions, pupil location and placing the incision farthest from the limbus as possible [6,7,11].

Intacs are reversible, well-tolerated and exchangeable. They provide flexibility to improve outcomes for individual patients, should the desired effect not be achieved with initial selection of inserts thickness. Modulation of postoperative outcomes with Intacs exchange warrant more studies. Use of a portable corneoscope or operating microscope-mounted topography unit may be valuable for refining the corrective effect achieved by Intacs inserts in individual cases. Refractive adjustments can be made during the procedure by replacing the original Intacs inserts choice with a thicker or a thinner one [6,37,40].

## CONCLUSION

The behavior of keratoconus tissue in the natural state and after ring implantation is not fully understood “no keratoconus eye is the same”. However, clinical and refractive data presented in this study show that Intacs, for the management of keratoconus, can be of great help in carefully-selected patients where the objectives are fully explained.

Intacs SK delay or stop the progression of the cone in selected cases. In fact, time is in favor of intacs as we noticed a decrease in the cone size postoperatively with the passage of time. Intacs help to shift from hard to soft contact lenses are reversible, well tolerated, safe and stable.

We have identified trends to decrease both lower and higher order aberrations in patients implanted with intacs as measured by OPD scan. Further studies and longer term follow-up are needed to evaluate Intacs for correction of keratoconus in subjects who can still tolerate contact lenses but would like to try an alternative treatment, as well as other applications for Intacs inserts. 

## DISCLOSURE

Conflicts of Interest: None declared.

## References

[1] Charlo FL, Joseph FF, Paolo MP (2002). Intrastromal corneal ring segments.

[2] Rabinowitz YS (1998). Keratoconus. Surv Ophthalmol.

[3] Brierly SC, Izquierdo L Jr, Mannis MJ (2000). Penetrating keratoplasty for keratoconus. Cornea.

[4] Barraquer JI (1949). Queratoplastia refractiva estudios e informaciones oftalmlógicas.

[5] Tuft SJ, Moodaley LC, Gregory WM, Davison CR, Buckley RJ (1994). Prognostic factors for the progression of keratoconus. Ophthalmology.

[6] Ibrahim TA, Sociedad Española de cirugía ocular implanto- refractiva (2005). Procedimientos quirúrgicos alternativos a la queratoplastia: Intacs, anillos intracorneales, Queratoplastia conductiva. Queratoplastia: Aspectos Refractivos.

[7] Carlo FL, Joseph FF, Paolo MP, Fabiano (2002). Preparation for surgery and surgical technique. Intrastromal corneal ring segments.

[8] Ibrahim TA ( 2003). Keratoconus, experience with INTACS. Course director presented at: Sociedad Espanola De Oftalmologia.

[9] Ibrahim TA (2006). Intacs in keratoconus: 5-year results.

[10] Ibrahim TA (2006). Intracorneal rings in keratoconus management.

[11] Colin J, Cochener B, Savary G, Malet F (2000). Correcting keratoconus with intracorneal rings. J Cataract Refract Surg.

[12] Chou B, Boxer Wachler BS (2000). Intacs for a keratocone: a promising new option?. Opt J Rev Optom.

[13] Boxer Wachler BS, Christie JP, Chandra NS, Chou B, Korn T, Nepomuceno R (2003). Intacs for keratoconus. Ophthalmology.

[14] Colin J (2006). European clinical evaluation: use of Intacs for the treatment of keratoconus. J Cataract Refract Surg.

[15] Rabinowitz YS, Klyce S, Krachmer J, Nordan L, Rowsey J, Sugar J, Wilson S, Binder P, Damiano R, McDonald M, Neuman A, Seiler T, Thompson K, Wyzinski P (1992). Keratoconus, videokeratography, and refractive surgery. Refract Corneal Surg.

[16] Colin J, Cochener B, Savary G, Malet F, Holmes-Higgin D (2001). INTACS inserts for treating keratoconus: one-year results. Ophthalmology.

[17] Maguire LJ, Bourne WM (1989). Corneal topography of early keratoconus. Am J Ophthalmol.

[18] Rabinowitz YS (1995). Videokeratographic indices to aid in screening for keratoconus. J Refract Surg.

[19] Langenbucher A, Gusek-Schneider GC, Kus MM, Huber D, Seitz B (1999). [Keratoconus screening with wave-front parameters based on topography height data]. Klin Monbl Augenheilkd.

[20] Baïkoff G, Maia N, Poulhalec D, Fontaine A, Giusiano B (1999). Diurnal variations in keratometry and refraction with intracorneal ring segments. J Cataract Refract Surg.

[21] Alió JL, Shabayek MH, Belda JI, Correas P, Feijoo ED (2006). Analysis of results related to good and bad outcomes of Intacs implantation for keratoconus correction. J Cataract Refract Surg.

[22] 22- Güell JL, Velasco F, Sánchez SI, Gris O, Garcia-Rojas M (2004). Intracorneal ring segments after laser in situ keratomileusis. J Refract Surg.

[23] Gomez L, Chayet A (2001). Laser in situ keratomileusis results after intrastromal corneal ring segments (Intacs). Ophthalmology.

[24] Rodriguez-Prats J, Galal A, Garcia-Lledo M, De La Hoz F, Alió JL (2003). Intracorneal rings for the correction of pellucid marginal degeneration. J Cataract Refract Surg.

[25] Barbara A, Shehadeh-Masha'our R, Garzozi HJ (2004). Intacs after laser in situ keratomileusis and photorefractive keratectomy. J Cataract Refract Surg.

[26] Mularoni A, Torreggiani A, di Biase A, Laffi GL, Tassinari G (2005). Conservative treatment of early and moderate pellucid marginal degeneration: a new refractive approach with intracorneal rings. Ophthalmology.

[27] Alió J, Salem T, Artola A, Osman A (2002). Intracorneal rings to correct corneal ectasia after laser in situ keratomileusis. J Cataract Refract Surg.

[28] Maguire LJ, Klyce SD, McDonald MB, Kaufman HE (1987). Corneal topography of pellucid marginal degeneration. Ophthalmology.

[29] Kymionis GD, Siganos CS, Kounis G, Astyrakakis N, Kalyvianaki MI, Pallikaris IG (2003). Management of post-LASIK corneal ectasia with Intacs inserts: one-year results. Arch Ophthalmol.

[30] Lovisolo CF, Fleming JF (2002). Intracorneal ring segments for iatrogenic keratectasia after laser in situ keratomileusis or photorefractive keratectomy. J Refract Surg.

[31] Sugar A (2002). Ultrafast (femtosecond) laser refractive surgery. Curr Opin Ophthalmol.

[32] Schanzlin DJ, Abbott RL, Asbell PA (2001). Two-year outcomes of intrastromal corneal ring segments for the correction of myopia. Ophthalmology.

[33] Nose' W, Neves RA, Schnzlin DJ, Belfort R Jr (1993). Intrastromal corneal ring - one-year results of first implants in humans: a preliminary nonfunctional eye study. Refract Corneal Surg.

[34] Bourcier T, Borderie V, Laroche L (2003). Late bacterial keratitis after implantation of intrastromal corneal ring segments. J Cataract Refract Surg.

[35] Ertan A, Bahadir M (2006). Intrastromal ring segment insertion using a femtosecond laser to correct pellucid marginal degeneration. J Cataract Refract Surg.

[36] Colin J, Velou S (2002). Utilization of refractive surgery technology in keratoconus and corneal transplants. Curr Opin Ophthalmol.

[37] Clinch TE, Lemp MA, Foulks GN, Schanzlin DJ (2002). Removal of INTACS for myopia. Ophthalmology.

[38] Chan SM, Khan HN (2002). Reversibility and exchangeability of intrastromal corneal ring segments. J Cataract Refract Surg.

[39] STONE W Jr, HERBERT E (1953). Experimental study of plastic material as replacement for the cornea; a preliminary report. Am J Ophthalmol.

[40] KRWAWICZ T (1961). New plastic operation for correcting the refractive error of aphakic eyes by changing the corneal curvature. Preliminary report. Br J Ophthalmol.

